# Resveratrol Counteracts Insulin Resistance—Potential Role of the Circulation

**DOI:** 10.3390/nu10091160

**Published:** 2018-08-24

**Authors:** Rachel H. X. Wong, Peter R. C. Howe

**Affiliations:** 1 School of Biomedical Sciences and Pharmacy, Clinical Nutrition Research Centre, University of Newcastle, Callaghan, NSW 2308, Australia; rachel.wong@newcastle.edu.au; 2 Institute for Resilient Regions, University of Southern Queensland, Springfield Central, QLD 4300, Australia

**Keywords:** diabetes, insulin resistance, microvascular function, vasoactive nutrients, resveratrol

## Abstract

Pre-clinical data and human trials indicate that resveratrol supplementation may help to counteract diabetes. Several mechanisms of action have been proposed to explain its metabolic benefits, including activation of sirtuins and estrogen receptors (ER) to promote glucose transporter type-4 (GLUT4) translocation and increase glucose uptake. Resveratrol can also enhance vasodilator function, yet the possibility that this action might help to alleviate insulin resistance in type-2 diabetes mellitus has received little attention. In this brief review we propose that, by restoring impaired endothelium-dependent vasodilatation in insulin resistant individuals resveratrol increases blood perfusion of skeletal muscle, thereby facilitating glucose delivery and utilization with resultant improvement of insulin sensitivity. Thus, circulatory improvements by vasoactive nutrients such as resveratrol may play a role in preventing or alleviating insulin resistance.

## 1. Does Resveratrol Improve Insulin Sensitivity? Evidence from Clinical Trials

The search for naturally occurring compounds that may help to prevent type 2 diabetes mellitus (T2DM) is gaining increasing attention. Whilst insulin resistance is a risk factor for developing T2DM, there are no specific pharmaceutical interventions to counteract insulin resistance, and only general diet and physical activity recommendations, for which adherence is poor and individual outcomes vary greatly. *Trans*-resveratrol, a stilbene found in edible plants such as berries, cocoa, peanut skins and red grapes, and therefore in red wine, is one promising candidate bioactive that has been recently identified with the potential to counteract (i.e., prevent as well as treat) insulin resistance. It has been shown to have many important physiological actions, primarily attributable to the activation of NAD-dependent histone deacetylases (sirtuins). However, with a similar molecular structure to estradiol (E_2_) and binding affinity for estrogen receptors (ER), it can also be considered as a phytoestrogen [1,2]. Interest in this compound was stimulated by a link between red wine consumption and lower cardiovascular disease mortality in the French population (the French Paradox), with resveratrol identified as a potential primary mediator. As a result, potential cardiovascular benefits of resveratrol have been extensively studied [3,4]. 

Insulin resistance, the primary abnormality of and risk factor for T2DM, is characterized by reduced insulin-mediated glucose uptake in skeletal muscle, liver and adipose tissue in people with normal glucose tolerance. In healthy individuals, a postprandial rise in glucose triggers pancreatic secretion of insulin which activates glucose transporter type-4 (GLUT4) transporters (highly expressed in muscles) to facilitate glucose uptake into muscle cells, liver and adipocytes for energy storage, whilst inhibiting the rate of glycogen breakdown in the liver [5,6]. Moreover, insulin can influence the rate of glucose uptake in skeletal muscle and adipose tissue by acting on the endothelium to recruit unperfused capillaries and increase blood flow [7]. Within one to three hours, the blood glucose level starts to decline and β-cells reduce insulin secretion. Because skeletal muscles are major sites for glucose uptake and storage, the vasodilator effects of insulin in skeletal muscle is of primary importance in this review. Insulin resistance, initiated by ageing, excessive dietary fat or glucose intake, or adiposity, can concertedly disrupt endothelial function, which can lead to impaired endothelium-dependent vasodilatation, reduced tissue perfusion and, therefore, decreased glucose disposal, triggering a self-perpetuating cycle of hyperinsulinaemia and hyperglycaemia [8,9]. The Homeostatic Model for Assessment of Insulin Resistance (HOMA-IR) is a validated algorithm that uses the product of fasting blood glucose and insulin to assess the extent to which the glucose-insulin feedback loop is disrupted [10]. 

Interest in and evidence for the potential of resveratrol to counteract T2DM by improving insulin sensitivity has grown. In a meta-analysis of 11 intervention trials conducted in T2DM [11], two showed that resveratrol could benefit T2DM by reducing fasting glucose, insulin, HOMA-IR and/or glycated haemoglobin (HbA1c). Movahed et al. found a 50% reduction in HOMA-IR and 14% improvement in HbA1c in T2DM adults following supplementation of 1000 mg/day for 45 days [12], with similar findings by Bhatt et al. [13]. Likewise, in the absence of a glucose-lowering effect, insulin levels were reduced by 5% in T2DM patients with periodontal disease following 4 weeks of 480 mg/day resveratrol [14]. However, a recent meta-analysis by Zhu et al. of nine studies performed in T2DM was only able to confirm a beneficial effect of resveratrol for reducing HOMA-IR [15], while other clinical studies examining the influence of resveratrol on diabetes biomarkers (i.e., insulin sensitivity and glucose tolerance) have been inconclusive, making the interpretation of results difficult [16,17]. It was argued that there may be an optimal dose range for metabolic effects since Brasyno et al. and Goh et al. did not find changes in insulin concentrations in T2DM patients receiving resveratrol doses of 10 mg/day and 3000 mg/day respectively [18,19]. A recent study by Timmers et al. [20] randomised obese volunteers with well-controlled T2DM to placebo or resveratrol (150 mg/day) for 30 days to evaluate insulin sensitivity via hyperinsulinaemic-euglycaemic clamps. However, hepatic and peripheral insulin sensitivity were unaffected by resveratrol, despite an ex vivo increase in muscle mitochondrial function and reduction of systolic blood pressure. The latter finding supports the robust benefits of resveratrol for cardiovascular function [3]. Nonetheless, it was argued that the trial participants were using a relatively high dose of metformin, which resulted in higher plasma levels of dihydro-resveratrol, a metabolite of resveratrol, suggesting a greater breakdown of resveratrol that may have affected its bioavailability. In addition, there is no evidence for insulin lowering in T2DM patients with advanced complications such as foot ulcers [21]. However, this study was not powered to detect changes in diabetic biomarkers. The potential for resveratrol to counteract severe T2DM is yet to be confirmed.

While Liu et al. concluded no significant antidiabetic benefits of resveratrol for non-T2DM adults in their meta-analysis [11], some clinical studies have shown otherwise. Witte et al. [22] found that resveratrol supplementation for six months reduced HbA1c in overweight but otherwise healthy older adults. In the same population, Timmers et al. observed a modest 14% insulin-lowering effect with 150 mg/day of resveratrol for 30 days. The improvement in insulin sensitivity was attributed to the enhancement of mitochondrial function in skeletal muscle and reduction in triglyceride and leptin levels, thereby enhancing substrate utilisation efficiency [23]. In contrast, a four-week trial using the same dose (150 mg/day) failed to show changes in lipids, inflammatory markers or glucose metabolism in overweight and slightly obese older men and women [24]. Similarly, six weeks of 2–3 g/day of resveratrol failed to change glucose tolerance or insulin sensitivity in older glucose-intolerant adults without T2DM [16]. Arguably the high resveratrol dose may have adversely affected insulin action via excessive activation of peroxisome proliferator-activated receptor-γ coactivator-1α (PGC1α), a central regulator of mitochondrial biogenesis and metabolism [25]. However, this finding contradicts a study in metabolic syndrome patients that found significant reductions in the area-under-the-curve of the postprandial rise in insulin concentration following 90 days of resveratrol treatment at 1500 mg/day [26]. 

The purpose of this review is to outline possible mechanisms by which resveratrol could improve insulin sensitivity, including the effects of human sirtuin 1 (SIRT1) activation on substrate utilisation. Given the conflicting clinical data on its metabolic effects and the emerging evidence that resveratrol can improve circulatory function [16,27], we hypothesise an additional mechanism by which resveratrol may help to counteract T2DM, viz. enhancing microvascular perfusion of skeletal muscle to facilitate glucose uptake and utilization. 

## 2. Mechanisms by Which Resveratrol May Improve Insulin Sensitivity in Skeletal Muscle

### 2.1. Human Sirtuin 1 (SIRT1) and AMP-Activated Protein Kinase (AMPK) Activation

Since its discovery, resveratrol has been shown to exert multifaceted effects on mitochondrial function, bone metabolism, cancer and neurodegenerative disease [28,29], the most consistent being its activation of sirtuins, which may also increase lifespan [30]. The activation of SIRT1 by resveratrol, resulting in increased in AMP-activated protein kinase (AMPK) activity, is thought to be a panacea for preventing age-induced diseases, including diabetes [25,31]. AMPK is expressed in various tissues, e.g., brain, liver, skeletal muscle and adipocytes [32]. Skeletal muscle is a major site for glucose uptake and glycogen synthesis (~80%) [33]. The postprandial elevation of blood glucose triggers insulin release, which suppresses endogenous glucose production (breakdown of glycogen) in muscle cells and activates GLUT4 transporters (highly expressed in muscles) to facilitate uptake of glucose into muscle cells for glycogen synthesis, thereby reducing blood glucose concentration [5,6]. As a master regulator of metabolism, the activation of AMPK upregulates mitochondrial biogenesis, inhibiting triglyceride synthesis and stimulating glucose uptake and fatty acid oxidation in the skeletal cells, which in turn improves insulin sensitivity [32,34].

Resveratrol has been shown to stimulate AMPK activity in hepatocytes [35], skeletal muscle cells [36], and neurons [37]. In insulin-resistant mice treated with resveratrol for 12 weeks, improvements in insulin-sensitivity and glucose tolerance were accompanied by upregulation of SIRT1 protein in liver and soleus muscle and consequent AMPK activation [38]. The anti-hyperglycaemic effect of chronic resveratrol treatment has been shown to improve whole-body and tissue specific insulin-stimulated glucose uptake in insulin-resistant rats on a high cholesterol-fructose diet by stimulating GLUT4 translocation to the cell membrane and insulin receptor phosphorylation in soleus muscles [2]. In diabetic men, resveratrol supplementation (3000 mg/day for 12 weeks) upregulated GLUT4 expression in skeletal muscle by enhancing SIRT1 expression and AMPK phosphorylation. However, these improvements did not translate into reductions of HbA1c or HOMA-IR [19]. It is still unclear whether SIRT1–AMPK activation by resveratrol is linked to improved insulin sensitivity in humans. 

### 2.2. Increased Glucose Uptake via Activation of Estrogen Receptors (ER)

ER, particularly ER-α, have recently been recognized to play a prominent role in modulating glucose disposal, primarily through effects on several proteins of the insulin-signaling pathway and on expression and translocation of GLUT4 [39]. GLUT4 expression in skeletal muscle cell membranes also depends on activation of ER-α to stimulate phosphorylation of protein kinase-B (Akt) and AMPK, for translocation of GLUT4 [40]. With a higher affinity for ER-α than ER-β, resveratrol has been shown to bind to ER on endothelial cells to concomitantly improve vasodilatation and stimulate skeletal muscle glucose uptake [41,42,43]. Inhibition of ER markedly suppressed resveratrol-induced muscle glucose uptake [2]. In human umbilical vein endothelial cells that express both ER-α and ER-β, a single dose of resveratrol can activate endothelial nitric oxide synthase (eNOS) to rapidly stimulate vasodilatation [44]. Furthermore, under euglycaemic-hyperinsulinaemic conditions, resveratrol treatment can induce ER-phosphorylation but not ER protein expression, supporting the view that the metabolic actions of resveratrol are mediated via ER activation [2]. 

Compared with resveratrol or insulin treatment alone, combined administration of resveratrol and insulin synergistically increased skeletal muscle glucose uptake and GLUT4 translocation in streptozotocin-induced diabetic rats [32]. However, this benefit was not seen with resveratrol and ER-agonist (E_2_) treatment, suggesting that resveratrol and E_2_ act on the same signaling pathway to enhance muscle glucose uptake. It can be noted that there was a dose-dependent reduction in plasma glucose levels in the rats; however, when the maximally effective resveratrol dose (0.5 mg/kg) was reached, no further benefit was observed with increasing doses. This observation has important implications for dosing in human studies. 

### 2.3. Improving Glucose Utilisation by Increasing Blood Flow in Skeletal Muscle

There is a mutual interaction between metabolic dysregulation and endothelial dysfunction in the development of insulin resistance. Indeed, the endothelium can also become insulin-resistant such that insulin-mediated vasodilatation is impaired [45]. Yet the role of microvascular dysfunction as a possible antecedent of insulin resistance that also synergistically contributes to cardiovascular morbidity and end-organ damage in T2DM has received little attention [46], even though Julius et al. had hypothesized a hemodynamic link between insulin resistance and hypertension 30 years ago [47]. The IMPORTANCE of a healthy circulation cannot be understated, particularly the responsiveness of the endothelium to enable arterioles to rapidly dilate or constrict in response to demands; this is vital for survival and the optimal functioning of tissues. The loss of endothelium-dependent vasodilatation caused by ageing, adiposity, excessive dietary fat and glucose intake or any other pathological influence marks the initial stage of vascular dysfunction. It is primarily mediated by the incapacity of vascular endothelial cells to synthesize and release the endogenous vasodilator, nitric oxide, giving rise to increased vascular resistance and causing impaired tissue perfusion. Consequently, this can lead to the suboptimal delivery of glucose and insulin to skeletal muscle, leading to a self-perpetuating cycle of hyperinsulinaemia and hyperglycaemia that ultimately results in glucose intolerance and exacerbates the endothelial dysfunction [9,48,49]. This impairment leads to arterial hypertension, capillary rarefaction (reduction in the density of capillaries) and tissue ischaemia, resulting in end-organ damage as in diabetic retinopathy. 

**Figure 1:**
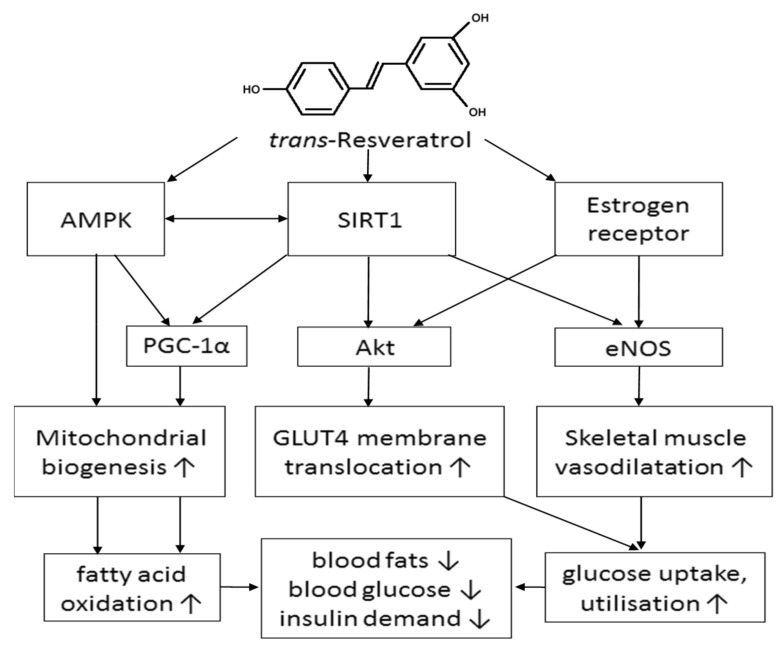
Multiple mechanisms by which resveratrol may counteract insulin resistance: activation of AMP-activated protein kinase–human sirtuin 1 (AMPK–SIRT1) pathways to enhance mitochondrial biogenesis and fatty acid oxidation; SIRT1 activation can also promote glucose transporter type-4 (GLUT4) translocation into cell membranes to facilitate glucose uptake; binding of resveratrol to estrogen receptors can enhance GLUT4 translocation. Moreover, we propose that both SIRT1 and estrogen receptor activation by resveratrol can increase endothelial nitric oxide synthase (eNOS) activity to augment blood flow in skeletal muscle, thereby further enhancing glucose delivery, uptake and substrate utilisation for energy production and reducing circulating blood glucose and insulin levels. Akt: protein kinase-B. PGC1α: peroxisome proliferator-activated receptor-γ coactivator-1α.

The mechanisms through which resveratrol may improve insulin sensitivity in T2DM are complex, encompassing reduced adiposity and changes in gene expression and activity of key enzymes [19,23]. Additionally, resveratrol may act to restore endothelium-dependent vasodilator function, thereby counteracting the vasoconstriction caused by hyperinsulinaemia. In insulin-resistant adults, excessive insulin levels promote vasoconstriction and vascular remodeling to create an ischaemic environment for tissues [8]. It is well established that resveratrol improves eNOS function by enhancing its expression and activity, resulting in increased nitric oxide bioavailability for vasodilatation, and prevents eNOS from uncoupling to reduced oxidative stress [50]. Furthermore, the simultaneous activation of SIRT1-AMPK and ER pathways by resveratrol can also indirectly increase eNOS expression and activity [50]. Hence, it is plausible that resveratrol-induced improvement in vasodilator capacity may restore perfusion to further promote glucose uptake in skeletal muscles, thereby reducing insulin demand (Figure 1). 

We were the first to demonstrate a dose-dependent increase in flow-mediated dilatation of the brachial artery during functional hyperaemia (FMD) following resveratrol consumption in mildly hypertensive and overweight adults [51]. FMD is a common method of assessing vasodilatory capacity in the systemic circulation and is closely associated with risk factors for cardiovascular disease [52]. Reduced FMD is an early marker of vascular pathology in obese individuals which precedes the loss of systemic arterial compliance and is linked to poor oxidative capacity in skeletal muscle [53]. Similarly, FMD is halved in T2DM compared to non-T2DM [54]. Chronic resveratrol supplementation (75 mg/day for 6 weeks) reversed the impairment of systemic vasodilator function in overweight/obese adults [27]. Interestingly, the magnitude of improvement in FMD was inversely proportional to their baseline vasodilator capacity, suggesting that mild endothelial dysfunction is reversible with resveratrol and that future intervention studies should select their subjects appropriately. A recent study [53] has confirmed the acute vasodilator benefit of a single 300 mg dose of resveratrol in treated hypertensive adults, aged 45–65 years, with poor FMD. They observed a greater increase of FMD following resveratrol consumption in adults with elevated low density lipoprotein cholesterol, emphasizing the potential for resveratrol to counteract endothelial dysfunction. Interestingly, they also found a significant 70% improvement of FMD in women but not in men, who had an 11% improvement of FMD [55]. This finding is consistent with the preclinical evidence that resveratrol, as a phytoestrogen, can also exert vasodilator effects through ER [2]. 

The vasodilator benefit of resveratrol extends to ameliorating microvascular dysfunction in the cerebral vessels of adults with T2DM, who have reduced cerebral perfusion, impaired cognitive function, and are at greater risk of developing dementia [56,57]. Similar to the FMD technique used in the systemic vasculature, a consistent hyperaemia can be achieved in cerebral vessels such as the middle cerebral artery by inhaling 5% carbon dioxide mixed with 95% oxygen (Carbogen gas). The magnitude of increase in blood flow velocity monitored by transcranial Doppler ultrasound reflects the vessel’s capacity to dilate effectively [58]. We recently demonstrated that the lowest dose of resveratrol tested (75 mg) afforded the maximum increase in cerebral vasodilator responsiveness to both hypercapnic and cognitive provocation compared to 150 mg and 300 mg doses [59,60]. Furthermore, we were able to confirm that chronic supplementation for 14 weeks with 150 mg/day of resveratrol resulted in sustained improvements of cerebrovascular responsiveness in postmenopausal women [61]. Women following menopause are equally, if not more, vulnerable to chronic diseases such as cognitive impairment, T2DM and osteoporosis [62,63,64]; these conditions are partially attributable to poor circulatory function. Hence, the sex difference in responsiveness to resveratrol warrants further investigation, as it may have potential to counteract endothelial dysfunction that is accelerated by estrogen deficiency at menopause. 

A single dose of resveratrol is unlikely to reduce arterial blood pressure, as seen in our study [51] and others [55]. However with regular supplementation, the functional changes of the vasculature may translate into favorable outcomes for arterial blood pressure. A meta-analysis of six studies reported a –5.66 mmHg non-significant reduction in systolic blood pressure; diastolic blood pressure was however unaffected with resveratrol supplementation. The lack of a clear antihypertensive benefit with resveratrol is suggested to be dose-dependent; at least 150 mg/day appears necessary in overweight and obese adults with established metabolic disturbances [4]. 

To date, no studies have evaluated whether the improvement in vascular function with resveratrol is accompanied by enhancement of insulin sensitivity in adults with metabolic dysregulation. Nonetheless, similar vasoactive nutrients such as cocoa flavanols that are well known to have favorable effects on FMD [65] have been shown to improve insulin sensitivity in hypertensive patients with impaired glucose tolerance. Compared to flavanol-free white chocolate, 100 g/day of flavanol-rich dark chocolate for 15 days significantly improved insulin sensitivity, as measured by HOMA-IR, β-cell function and arterial blood pressure. In fact, the changes in insulin sensitivity and β-cell function directly correlated with FMD enhancements (*r* = 0.510) and blood pressure reduction [66]. Recently more bioactives have been shown to have promising effects. 

## 3. Conclusions

We have summarized the possible mechanisms by which resveratrol may act to improve insulin sensitivity, with particular emphasis on the role of the microcirculation to improve/delivery of glucose and fatty acids to skeletal muscles. These are summarized in Figure 1. Whilst the clinical trial evidence for metabolic health improvements with resveratrol is mixed, partly due to variations in dose levels used, subject selection, trial duration, present comorbidities and medication use, the positive effects of resveratrol on vascular health are consistent, emphasizing the potential importance of skeletal muscle perfusion in the prevention of T2D. The effectiveness of resveratrol for improving insulin sensitivity, mediated by SIRT1–AMPK, ER and eNOS activation, appears to be limited to overweight/obese/insulin resistant individuals with impaired endothelial function. In fact, clinical evidence supports the reversibility of endothelial dysfunction and the normalization of metabolic homeostasis. Further studies to identify the optimal resveratrol dose for improving, preventing and managing T2DM are urgently warranted. In view of the potential importance of the phytoestrogenic effects of resveratrol on glucose metabolism, including the improvement of skeletal muscle perfusion, sex differences in responsiveness to resveratrol treatment should also be evaluated.
